# First Draft Genome Sequences of Malaysian Clinical Isolates of *Corynebacterium diphtheriae*

**DOI:** 10.1128/genomeA.01670-16

**Published:** 2017-03-02

**Authors:** Norazah Ahmad, Shirley Yi Fen Hii, Mohd Khairul Nizam Mohd Khalid, Muhammad Adib Abd Wahab, Rohaidah Hashim, Soo Nee Tang, Yii Ling Liow, Hazwani Hamzah, Nurul Ain Dahalan, Valentinus Seradja

**Affiliations:** a Bacteriology Unit, Infectious Diseases Research Centre, Institute for Medical Research, Kuala Lumpur, Malaysia; b Molecular Diagnostics and Protein Unit, Specialised Diagnostics Centre, Institute for Medical Research, Kuala Lumpur, Malaysia

## Abstract

*Corynebacterium diphtheriae* has caused multiple isolated diphtheria cases in Malaysia over the years. Here, we report the first draft genome sequences of 15 Malaysia *C. diphtheriae* clinical isolates collected from the years 1981 to 2016.

## GENOME ANNOUNCEMENT

Diphtheria is a serious and potentially fatal infection caused by the Gram-positive bacterium, *Corynebacterium diphtheriae*. The clinical presentation is apparent with the formation of toxemic grayish pseudomembrane at the upper respiratory tract (1). In Malaysia, despite mass vaccination, there have always been sporadic cases related to nonvaccinated or incompletely vaccinated patients. Recently, there has been an increase in the number of diphtheria cases occurring in several states in Malaysia. To our knowledge, there have been no genome submissions of Malaysian *C. diphtheriae* isolates. We undertook the first attempt to sequence 15 clinical isolates of *C. diphtheriae* from Malaysia.

The 15 isolates of *C. diphtheriae* were confirmed following the method shown by Efstratiou et al. (2). The genomic DNA was extracted using a Gram-positive DNA purification kit (Epicentre, Madison, WI, USA) following the manufacturer’s protocol and a Nextera XT DNA sample preparation kit (Illumina, San Diego, CA, USA) was used to construct DNA libraries. The sequencing was performed using a whole-genome shotgun approach performed on an Illumina MiSeq system (Illumina, San Diego, USA) with 2 × 301 bp paired-end chemistry. Raw reads were preprocessed to remove adapter sequences and low-quality reads using Trimmomatic (v0.36) software (3).

*De novo* assembly of the reads was performed using IDBA-UD genome assembler (v1.0.9) (4) that generated contig numbers between 24 and 46 per genome with a total size ranging from 2.37 Mbp to 2.50 Mbp. The G+C content ranged from 53.4% to 53.6%. The protein-coding gene prediction performed using Prodigal (v2.60) (5) showed a range of 2,213 to 2,413 sequences per genome. The tRNAs and rRNAs were predicted using ARAGORN (v1.2.34) (6) and RNAmmer (v1.2) (7), respectively. The number of tRNA genes ranged from 100 to 104 while five rRNA genes were identified in all the genomes.

### Accession number(s).

The sequences of this whole-genome shotgun project have been deposited in DDBJ/ENA/GenBank under the accession numbers listed in Table 1.

**TABLE 1:** Genome assembly details and statistics

Isolate	NCBI BioSample no.	GenBank accession no.	Genome size (bp)	No. of contigs	*N*_50_ (bp)
rz252	SAMN05868145	MKYG00000000	2,450,113	35	158,453
rz319	SAMN05877636	MKYM00000000	2,495,722	27	200,191
rz356	SAMN05877637	MKYI00000000	2,476,729	42	183,724
rz358	SAMN05877638	MKYJ00000000	2,370,538	24	171,693
rz373	SAMN05877639	MKYK00000000	2,433,368	42	127,819
rz378	SAMN05877640	MKYL00000000	2,449,239	40	141,127
rz379	SAMN05877641	MKYH00000000	2,449,433	37	128,737
c20	SAMN05877642	MLBN00000000	2,471,211	37	158,285
c21	SAMN05877643	MKYN00000000	2,500,797	40	103,815
c110	SAMN05877644	MLBH00000000	2,466,694	37	181,540
c122	SAMN05877645	MLBI00000000	2,390,956	36	129,561
c123	SAMN05877646	MLBJ00000000	2,444,540	41	128,795
c325	SAMN05877647	MLBK00000000	2,472,120	43	137,577
c488	SAMN05877648	MLBL00000000	2,475,046	46	124,164
c517	SAMN05877649	MLBM00000000	2,464,057	34	157,136
